# Maximizing Health-Care Capacity in Response to COVID-19 Outbreak: Rapid Expansion Through Education by Health Emergency and Disaster Experts

**DOI:** 10.1017/dmp.2020.264

**Published:** 2020-07-27

**Authors:** Soichiro Kato, Yasuhiko Miyakuni, Yoshitaka Inoue, Yoshihiro Yamaguchi

**Affiliations:** Instructor of the Tokyo Disaster Medical Assistance Team, Tokyo, Japan; Chairperson of the Tokyo Disaster Medical Assistance Team, Tokyo, Japan; Disaster Medical Coordinator of the Tokyo Metropolitan Government, Tokyo, Japan

**Keywords:** disaster medicine, emergency medicine, disaster planning, emergency medical services, emergency preparedness

## Abstract

Delivering adequate health care in the setting of the ongoing pandemic is challenging. Due to coronavirus disease 2019 (COVID-19), the Tokyo Metropolitan government has been forced to expand their acute health-care capacity corresponding to infectious diseases within a short period. Responding to this situation, health emergency and disaster experts of the Tokyo Disaster Medical Assistance Team took the initiative in creating a brief education course. We established the course for expanding infectious disease care capacity by a dedicated hands-on lecture for health professionals who are unfamiliar with infectious disease care in ordinary circumstances. Our lecture included the typical course of COVID-19, use of personal protective equipment, environmental sterilization, medical-ward zoning, and safe caregiving. Hospitals that received customized lectures reported by means of a questionnaire that the lectures were well suited to their needs. Currently, the health-care system in Tokyo has increased its capacity to meet the demand and has not been affected by COVID-19. Our experience shows that health emergency and disaster experts can assist hospitals in crisis by providing educational materials.

As of May 4, 2020, Japan has reported 15,057 confirmed cases of novel coronavirus disease 2019 (COVID-19), including 510 COVID-19-related deaths.^1^ Approximately one-third of all confirmed cases (*n* = 4654) have occurred in the Tokyo metropolitan area,^2^ the most densely populated and highly centralized city in Japan. The number of reported cases continues to increase in some densely populated areas of Japan, and the country remains at risk of infection overshoot and health-care system breakdown. Since late March 2020, increasing the hospitals’ surge capacity for inpatient health care for COVID-19, including critical care, is a high-priority issue in the Tokyo metropolitan area. In this article, we reported a rapid acute health-care capacity expansion due to the COVID-19 outbreak in Japan, through a brief education course performed by health emergency and disaster experts.

## HEALTH-CARE CAPACITY FOR COVID-19 IN TOKYO

Delivering adequate health care despite the ongoing pandemic is challenging^3-5^ and requires a 3-stage strategy: providing easy-access medical consultations for patients with mild symptoms, maximizing hospitals’ capacity for safe and appropriate admission and care of patients with moderate symptoms, and strengthening critical-care capacity for severely ill patients. Japan’s acute health-care system has a 3-layer structure^6^ enabling it to meet the demands of each severity level. Tokyo metropolitan area responded during the early phase of the pandemic to the growing shortage of care capacity by preidentifying facilities that could be used for infectious disease treatment. Most of these predetermined facilities for infectious diseases (PFIDs) were intended to serve moderately and severely ill patients with a well-trained staff to respond to health emergencies. However, the total capacities of these facilities were too small for the pandemic. Most non-PFIDs, with more beds and larger capacities, identified to serve mildly and moderately ill patients are not intended to treat patients with infectious diseases under ordinary circumstances. Furthermore, the government recognized the need to increase the health-care system’s capacity to care for COVID-19 patients under particular situations, such as expectant women, children, patients with mental disorder or dementia, and patients receiving dialysis. Facilities intended to serve such patients are generally not familiar with infectious disease care, including personal protective equipment (PPE) use.

## RAPID CAPACITY EXPANSION THROUGH EDUCATION

To address this dilemma, the Tokyo Disaster Medical Assistance Team (DMAT) created and delivered a brief course, in early April 2020, for non-PFIDs on preparing for COVID-19 patient reception. The Tokyo DMAT, founded in 2004 to respond to health emergencies including natural disasters and mass casualty incidents, includes over a thousand acute health-care providers belonging to 25 Tokyo hospitals.^7-10^ In addition to ordinary teams composed of emergency physicians, acute care nurses, and logistics personnel, 5 of these hospitals also have chemical, biological, radiological, and nuclear specialist teams. These teams took the initiative in creating the course and delivering in-hospital lectures regarding clinical information, hospital management, and essential nursing points for the COVID-19 response. The development of this course was supported by the Tokyo Metropolitan Government and the Tokyo Medical Association.

The course instructed health professionals who were formerly unskilled in protection against infection to respond safely and effectively to COVID-19. Our primary topics were the typical course of COVID-19, PPE use, environmental sterilization, medical-ward zoning, and safe caregiving. The contents were determined after discussion between the disaster medicine specialists and infectious disease experts. The infectious disease experts ensured that the content was current and of high quality. Because the course creators understood the ability of local facilities based on their daily clinical activities, the educational intervention could be designed to provide the necessary information for attendees. We provided a leaflet on hospital preparedness and infection control response and delivered customized lectures on the ward of their response to health emergencies. The exigency required us to expediently create the course to eliminate the risk of instructors getting infected. During 5 d in early April 2020, we were able to deliver customized on-site lectures to 6 of the 42 hospitals that requested them. The course pedagogy was based on adult education and we performed problem-based learning such as tutorials led by a short introductory lecture. Therefore, these were flexible, depending on the needs of each attendee and hospital. The course agenda is shown in Table 1.

**TABLE 1:** Course Agenda of Basic Infection Control Methods Targeted at Non-predetermined Facilities for Infectious Diseases

Session	Time	Description of Course Experience
1	5 min	Introductory remarks from the course managers, government officials
2	5 min	Course introduction from the instructors
3	15 min	Rationale for safe response to COVID-19
		(1) Basic information of the infection route
		(2) Principle of PPE dressing and undressing
		(3) Surgical mask and N95 mask wearing
		(4) Principle of area zoning
		(5) Characteristics of COVID-19
		(6) Overview of the hospital preparedness for COVID-19
		(7) Environmental control and sterilization
		(8) Tips of risk reduction for medical professionals
4	15 min	Key points of treatment and nursing care for COVID-19 patients
		(1) Breathing problems and respiratory care
		(2) Cautions against performing invasive procedures
		(3) Cautions against performing laboratory and image examinations
		(4) Prevention of hospital infection
		(5) Avoidance of close contacts
		(6) Response to dead cases
5	30 to 60 min	Hands-on instruction
	(depending on the situation)	(1) Ward preparation for receiving COVID-19 patients
		(2) Flow of infected patients
		(3) PPE dressing and undressing
		(4) Case simulations
		(5) Other points and tips depending on each wards
6	30 min	Question/answer period

Abbreviations: PPE, personal protective equipment; COVID-19, coronavirus disease 2019.

Immediately afterward, the remaining 36 and several dozen hospitals were provided with instructional videos. Attended hospitals and its staff orally reported to metropolitan officials that the course was well suited to their needs. Furthermore, several hospitals started providing inpatient care to COVID-19 patients after the lecture. Our efforts have helped increase the capacities of Tokyo hospitals for COVID-19, including for the patients with particular situations.

Although the number of COVID-19 patients in Tokyo is still growing,^2^ the health-care system has increased its capacity to meet the demand and has not broken down. Our experience shows that health emergency and disaster experts can assist hospitals in crisis by providing educational materials. During a pandemic, collaboration between infectious disease experts and health emergency specialists enables prompt and effective support for frontline hospitals.
